# Profound Sinoatrial Arrest Associated with Ibrutinib

**DOI:** 10.1155/2017/7304021

**Published:** 2017-12-10

**Authors:** Kanupriya Mathur, Aditya Saini, Kenneth A. Ellenbogen, Richard K. Shepard

**Affiliations:** ^1^Department of Internal Medicine, Virginia Commonwealth University, Richmond, VA, USA; ^2^Department of Cardiac Electrophysiology, Virginia Commonwealth University, Richmond, VA, USA

## Abstract

**Background:**

Ibrutinib is a Bruton's tyrosine kinase (BTK) inhibitor approved for second-line treatment for mantle cell lymphoma (MCL), chronic lymphocytic leukemia (CLL), and Waldenström macroglobulinemia. Ibrutinib use has been linked to increased incidence of atrial fibrillation and hypertension in multiple studies. Other forms of cardiac toxicities have also been reported in isolated case reports. Bradycardia and asystole have not been associated with ibrutinib use in the past.

**Case Report:**

We present a case of a 76-year-old female with no prior cardiac history, who initiated treatment with ibrutinib for relapsing mantle cell lymphoma and was noted to have symptomatic bradycardia, greater than 20 second long pauses on her cardiac monitor requiring placement of a permanent pacemaker.

**Conclusion:**

This is the first case associating bradycardia and asystole with tyrosine kinase inhibitor use. Irreversible inhibition of certain cardioprotective tyrosine kinases has been a growing topic of research in oncology therapeutics.

## 1. Background

Ibrutinib (Imbruvica, Pharmacyclics, Inc.) is a Bruton's tyrosine kinase (BTK) inhibitor approved for use in patients with mantle cell lymphoma (MCL), chronic lymphocytic leukemia (CLL), and Waldenström macroglobulinemia. Ibrutinib use has been linked to increased incidence of atrial fibrillation and hypertension in multiple studies [1–4]. Other forms of cardiac toxicities have also been reported in isolated case reports [5]. We report a case of recurrent and profound sinus arrest in a patient treated with ibrutinib for MCL.

## 2. Case Report

A 76-year-old Caucasian female with a past medical history of mantle cell lymphoma (MCL), hypertension, and gastroesophageal reflux disease presented to the hospital with intractable nausea and vomiting of one week duration. With regard to treatment of her MCL, she had failed first-line treatment with bendamustine-rituximab following which she had received R-CHOP (rituximab, cyclophosphamide, vincristine, and prednisolone) chemotherapy. However, due to her advancing disease detected by positron emission tomography imaging and lymph node biopsy, she was started on ibrutinib 560 mg daily 11 days ago. She took the medication for 8 days after which she could not tolerate it due to intractable nausea and vomiting. She was unable to tolerate any of her other medications and quit taking all of them 5 days prior to admission. Her other home medications were reviewed, which included atorvastatin 20 mg daily, carvedilol 3.125 mg twice daily, citalopram 10 mg daily, and lisinopril 5 mg daily. While in the hospital, she continued to have nausea for two more days. During this time, her potassium (K+) level was low (range: 2.4–3.2 meq/L), and she received K+ replacement. In the first 48 hours of hospitalization, she experienced 2 episodes of brief syncope lasting a few seconds and sinus pauses (up to 6 seconds), which appeared to be vagally mediated, as they occurred immediately after an episode of retching or coughing. Her baseline 12-lead electrocardiogram showed normal sinus rhythm with normal PR interval and preexisting right bundle branch block with QRS duration of 138 milliseconds. She had no prior history of syncope, bradycardia, or any cardiac illness. She had no family history of sudden cardiac death. At the time of the event, she was not on any medications known to have a potential to cause bradycardia. Transthoracic echocardiogram revealed an ejection fraction of 60–65% and was otherwise normal.

**Figure 1:**
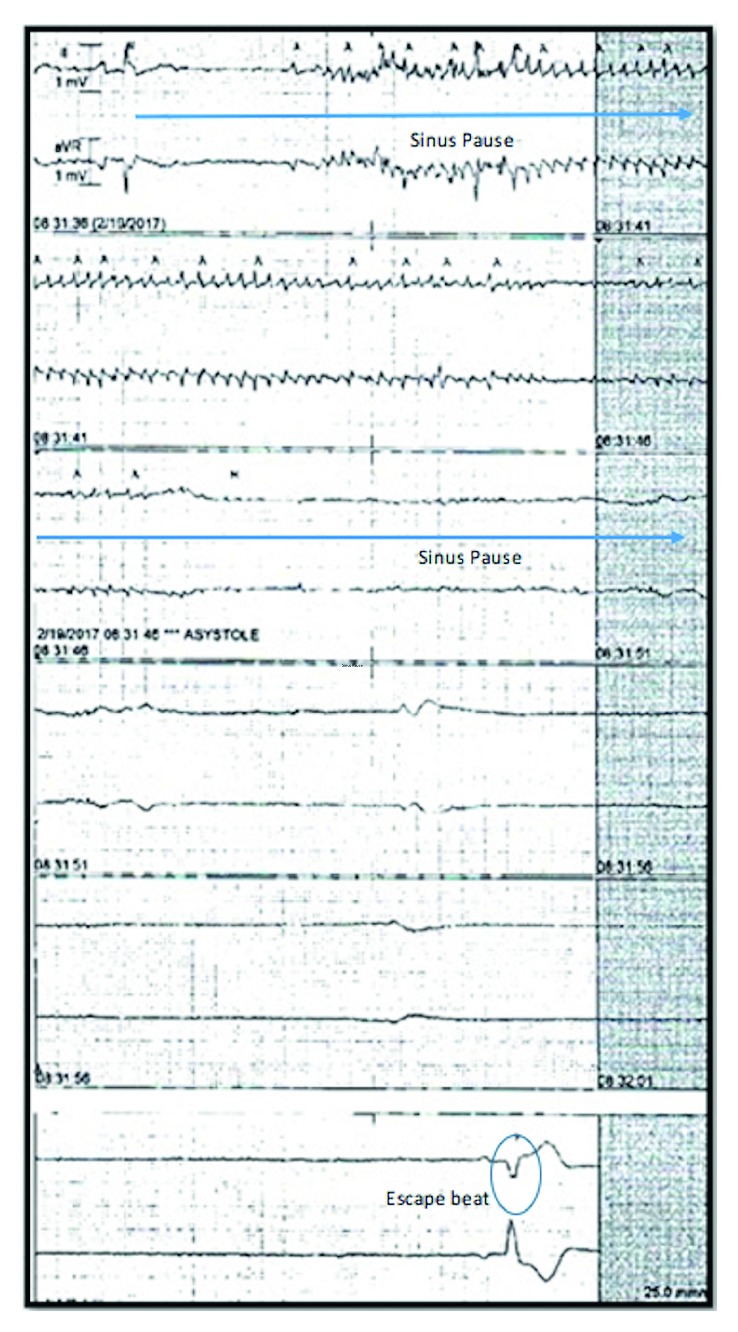
Telemetry recording from the time of syncopal event in our patient demonstrating sinoatrial arrest and a 26-second pause. Notice the absence of any atrial activity during the pause indicating this is sinus pause rather than AV block. The telemetry marks some tremor artifact during the event erroneously as atrial activity (A) which is another important finding to recognize.

By the third day of hospitalization, her symptoms had resolved and she was able to tolerate solid food with no difficulty. Her electrolytes were in normal range. However, she suddenly had a witnessed syncopal episode correlating with 26 seconds of sinoatrial arrest noted on telemetry (Figure 1). The episode was abrupt in onset. The patient was in bed and was talking to her nurse when she had this syncopal episode. She regained her baseline level of consciousness after the episode with no residual deficits. Cardiology consult was obtained, and a permanent pacemaker was planned. While awaiting pacemaker implantation, she developed two more symptomatic episodes of abrupt onset sinus pauses over next 24 hours without any trigger. A dual-chamber permanent pacemaker was implanted the next day. Patient was not given ibrutinib again.

## 3. Discussion

Ibrutinib is a Bruton's tyrosine kinase (BTK) inhibitor that has been approved as a second-line agent for refractory/relapsedMCL. In November 2013, the Food and Drug Administration (FDA) granted accelerated approval to ibrutinib for the treatment of patients with MCL who have received at least one prior therapy. This approval was based on the results of an international multicenter trial of 111 patients with previously treated MCL which demonstrated favorable efficacy of the medication [1]. The common adverse events reported are diarrhea, fatigue, nausea, vomiting, peripheral edema, dyspnea, constipation, upper respiratory tract infection, and pneumonia in addition to hematological adverse events like neutropenia, thrombocytopenia, anemia, and bleeding.

There has been growing interest in cardiac side effects of tyrosine kinase inhibitors. Ibrutinib use particularly has been linked with cardiac manifestations, but the commonly associated condition with this drug is atrial fibrillation. In one study, 6-month, 1-year, and 2-year cumulative incidences of AF in patients with MCL who were treated with ibrutinib were 5.6%, 7.2%, and 14.2%, respectively [3]. In trials of ibrutinib in CLL patients, AF incidence of 5–7.7% has been reported [4]. The RESONATE trial for chronic lymphocytic leukemia showed a 10-fold increase in atrial fibrillation in patients treated with ibrutinib as compared to ofatumumab [6]. Life-threatening ventricular arrhythmias and sudden cardiac death were recently reported in a compilation of cases by Lampson et al. [7]. Ibrutinib is an irreversible tyrosine kinase inhibitor. Although the mechanism of cardiac toxicities of ibrutinib remains uncertain, it is possible that this at least in part may be due to irreversible inhibition of certain protective tyrosine kinases which may have a key role in cardiac function and electrophysiological properties. The irreversible inhibition may also explain why manifestations can occur even when the drug is withdrawn. According to one hypothesis, ibrutinib inhibits the protective PI3k-Akt signaling pathway, which may play a role in cardiac side effects of the medication [8]. TEC pathway inhibition is another plausible mechanism that may contribute to ibrutinib's cardiac toxicity, as referred by Tang et al. in 2017 [9]. One case of new onset cardiomyopathy and ventricular tachycardia has been reported with ibrutinib use [8]. The exact spectrum of cardiac manifestations of the medication as well as relation to dose and duration of therapy remains to be studied.

A detailed cardiovascular safety report, published in the *Drug Safety* journal in 2015, describes the common cardiac effects associated with tyrosine kinase inhibitors (25 medications) approved for several oncologic indications. These include hypertension, pulmonary hypertension, bleeding, arterial and venous thrombosis, congestive heart failure, QT prolongation and associated arrhythmias, sudden death, and syncope. To our knowledge, this is the first case report of recurrent profound bradycardia due to paroxysmal sinoatrial arrest in a patient treated with ibrutinib. The authors would like to emphasize that it is possible our patient had previously undiagnosed sick sinus syndrome or autonomic dysfunction, which may have been the cause of sinus pauses and syncope. However, in light of recent use of ibrutinib in our patient, a medication that has been linked to cardiac side effects as well as with absence of any history of prior syncopal or presyncopal symptoms, the hypothesis that ibrutinib use resulted in profound depression of the sinus node or aggravated preexisting subclinical sinus node dysfunction has to be entertained.

## 4. Conclusion

With ever-expanding indications for chemotherapeutic agents and immunomodulators, the clinician must remain vigilant for unknown and rare systemic manifestations that may be associated with their use. Some of these effects may be potentially life-threatening. Our case highlights the need for further studies aimed at providing mechanistic insights into cardiac toxicities of ibrutinib.
